# Impact of genetic variants on clinical outcome after percutaneous coronary intervention in elderly patients

**DOI:** 10.18632/aging.202799

**Published:** 2021-03-12

**Authors:** Jung-Joon Cha, Jae Hyoung Park, Hyung Joon Joo, Soon Jun Hong, Tae Hoon Ahn, Byeong-Keuk Kim, WonYong Shin, Sung Gyun Ahn, JungHan Yoon, Yong Hoon Kim, Yun-Hyeong Cho, Woong Chol Kang, Weon Kim, Young-Hyo Lim, Hyeon Cheol Gwon, Woong Gil Choi, Do-Sun Lim

**Affiliations:** 1 Department of Cardiology, Cardiovascular Center, Korea University Anam Hospital, Korea University College of Medicine, Seoul, South Korea; 2 Division of Cardiology, Severance Cardiovascular Hospital, Yonsei University College of Medicine, Seoul, South Korea; 3 Division of Cardiology, Department of Internal Medicine, Soonchunhyang University Cheonan Hospital, Cheonan, South Korea; 4 Department of Cardiology, Yonsei University Wonju Severance Christian Hospital, Wonju, South Korea; 5 Division of Cardiology, Department of Internal Medicine, Kangwon National University School of Medicine, Chuncheon, South Korea; 6 Department of Internal Medicine, Hanyang University Myongji Hospital, Goyang, South Korea; 7 Department of Cardiology, Gachon University Gil Medical Center, Incheon, South Korea; 8 Department of Internal Medicine, Division of Cardiology, Kyung Hee University Hospital, Kyung Hee University School of Medicine, Seoul, South Korea; 9 Division of Cardiology, Department of Internal Medicine, Hanyang University College of Medicine, Seoul, South Korea; 10 Division of Cardiology, Department of Medicine, Samsung Medical Center, Sungkyunkwan University School of Medicine, Seoul, South Korea; 11 Division of Cardiology, Department of Internal Medicine, Konkuk University College of Medicine, Chungju, South Korea

**Keywords:** elderly, cytochrome P-450 CYP2C19, P2Y12 receptor gene polymorphism, coronary artery disease, clinical outcome

## Abstract

Elderly patients treated with percutaneous coronary intervention (PCI) have a higher risk of both ischemic and bleeding complications than younger patients. However, few studies have reported how genetic information of elderly patients treated with PCI affects clinical outcomes. We investigated the impact of genetic variants on clinical outcomes in elderly patients. Correlations between single-nucleotide polymorphisms (CYP2C19 and P2Y12 receptor gene G52T polymorphism) and clinical outcomes were analyzed in 811 elderly patients (≥75 years of age) from a prospective multicenter registry. The primary endpoint was a composite of myocardial infarction and death. Secondary endpoints were an individual event of death, cardiac death, myocardial infarction, stent thrombosis, target lesion revascularization, stroke, and major bleeding (Bleeding Academic Research Consortium ≥3). Regarding CYP2C19, patients with poor metabolizers had a significantly higher risk for the primary endpoint (hazard ratio [HR] 2.43; 95% confidence interval [95% CI] 1.12–5.24; p=0.024) and secondary endpoints (death and cardiac death). Regarding P2Y12 G52T, the TT group had a significantly higher occurrence of major bleeding than the other groups (HR 3.87; 95% CI 1.41–10.68; p=0.009). In conclusion, poor metabolizers of CYP2C19 and TT groups of P2Y12 G52T may be significant predictors of poor clinical outcomes in elderly patients.

## INTRODUCTION

The number of elderly patients treated with percutaneous coronary intervention (PCI) for coronary artery disease is increasing [1, 2]. However, elderly patients who undergo PCI due to complex clinical conditions, such as comorbidities, show poor clinical outcomes [3, 4]. Elderly patients who undergo PCI have a higher risk of ischemic events and bleeding complications than younger patients and—consequently—a higher mortality rate [5]. Numerous general population studies have been conducted on the optimal dual antiplatelet therapy (DAPT) strategy to reduce ischemic and bleeding risk [6]. In addition, a tailored DAPT strategy based on genetic variation has also been proposed [7–9]. However, few studies have addressed tailored DAPT based on genetic variation in elderly patients. This multicenter, prospective, observational study investigated the associations between genetic variants and clinical outcomes in elderly patients after PCI.

## RESULTS

### Baseline characteristics and prevalence of genetic variants in elderly patients

A total of 811 elderly patients (≥75 years of age) were enrolled in this study (Supplementary Figure 1 and Supplementary Table 1). The mean age was 79.1±3.6, and 53% were men. Index PCI due to acute coronary syndrome was performed in 452 (55.7%) patients, and 132 (16.3%) patients had multivessel disease. A total of 545 second-generation drug-eluting stents (DESs) with durable polymers were implanted in the enrolled patients (67.2%). In addition, third-generation DESs with biodegradable polymers were implanted in 27% of patients. Bare metal stents and first-generation DESs are rarely used (0.5%). The mean duration of DAPT (aspirin plus clopidogrel) was 321.4±89.5 days, and 80% of patients maintained DAPT for >1 year. The prevalence of CYP2C19 and P2Y12 G52T variants was 63% and 26%, respectively. CYP2C19 variants were classified into three groups according to their phenotypes: normal metabolizer (*1/*1), intermediate metabolizer (*1/*2 and *1/*3), and poor metabolizer (*2/*2, *2/*3, and *3/*3) (Supplementary Table 1).

### CYP2C19

According to the clinical pharmacogenetics implementation consortium guidelines for the CYP2C19 genotype [10], we classified the normal and intermediate metabolizers into one group and poor metabolizers into a second group. There were no statistically significant between-group differences in baseline characteristics, including the presentation of acute coronary syndrome and PRECISE-DAPT (Predicting Bleeding Complication in Patients Undergoing Stent Implantation and Subsequent Dual Antiplatelet Therapy) score (Table 1). The two groups had no significant differences in lesion characteristics and genetic variations (Supplementary Table 2). The two groups had similar discharge medications, without significant differences in in-hospital events. During the platelet function test, the poor metabolizers had a higher P2Y12 reaction unit (PRU) than the other group (269.9±82.8 vs. 234.0±74.8, respectively; p<0.001). However, no significant between-group differences were observed for DAPT duration (Supplementary Table 3).

**Table 1:** Baseline characteristics and genetic variation according to CYP2C19 variant in elderly patients.

	**NM or IM**	**PM**	**p-value**
	**(n=694)**	**(n=117)**	
**Male sex**	376 (54.2%)	57 (48.7%)	0.320
**Age (years)**	79.1±3.7	79.1±3.4	0.835
**Body-mass index (kg/m^2^)**	23.7±3.4	23.7±3.1	0.978
**Current smoker**	71 (10.2%)	16 (13.7%)	0.341
**Hypertension**	508 (73.2%)	86 (73.5%)	1.000
**Diabetes mellitus**	242 (34.9%)	36 (30.8%)	0.448
**Hypercholesterolemia**	211 (30.4%)	41 (35.0%)	0.371
**Previous MI**	46 (6.6%)	5 (4.3%)	0.444
**Previous PCI**	106 (15.3%)	20 (17.1%)	0.715
**Previous CABG**	9 (1.3%)	2 (1.7%)	1.000
**Previous CVA**	75 (10.8%)	16 (13.7%)	0.453
**Congestive heart failure**	44 (6.3%)	8 (6.8%)	1.000
**Chronic kidney disease**	49 (7.1%)	4 (3.4%)	0.203
**Familial history of CAD**	23 (3.3%)	5 (4.3%)	0.801
**Anemia**	329 (47.4%)	53 (45.3%)	0.692
**Presentation with ACS**	382 (55.0%)	70 (59.8%)	0.366
**PRECISE-DAPT score**	25.9 ± 9.3	25.8 ± 10.3	0.889
**- High risk**	328 (47.3%)	50 (42.7%)	0.384
**P2Y12 G52T (rs6809699)**			0.238
**- GG**	519 (74.8%)	79 (67.5%)	
**- GT**	153 (22.0%)	34 (29.1%)	
**- TT**	22 (3.2%)	4 (3.4%)	

Values are expressed as the number of patients (%) or mean (SD). ACS, acute coronary syndrome; CABG, coronary artery bypass graft; CAD, coronary artery disease; CVA, cerebrovascular accident; IM, intermediate metabolizer; MI, myocardial infarction; NM, normal metabolizer; PCI, percutaneous coronary intervention; PM, poor metabolizer.

### P2Y12 platelet adenosine 5′-diphosphate (ADP) receptor gene G52T polymorphism

There were no statistical differences among the variants in baseline characteristics, including presentation of acute coronary syndrome, PRECISE-DAPT score, and genetic variations except history of hypertension (Table 2 and Supplementary Table 4). However, The TT group of patients experienced significantly more in-hospital strokes (TT vs. GT vs. GG; 3.8% vs. 0.5% vs. 0.0%, p<0.001, respectively; Supplementary Table 5) and in-hospital bleeding compared with the other group (19.2% vs. 2.1% vs. 4.5%, p=0.001; Supplementary Table 5) despite no significant between-group differences in discharge medications and PRU (254.2±60.8 vs. 239.4±78.5 vs. 238.4±77.2, p=0.593). In addition, the overall duration of DAPT (346.5±39.1 vs. 324.4±89.4 vs. 319.4±91.0, p=0.280) and the rate of DAPT maintenance for >1 year (85% vs. 83% vs. 78%, p=0.233) were not significantly different between the groups.

**Table 2:** Baseline characteristics and genetic variation according to P2Y12 platelet adenosine 5′-diphosphate receptor G52T polymorphism in elderly patients.

	**GG**	**GT**	**TT**	**p-value**
	**(N=598)**	**(N=187)**	**(N=26)**	
**Male sex**	312 (52.2%)	110 (58.8%)	11 (42.3%)	0.145
**Age (years)**	79.2±3.7	79.0±3.4	78.6±3.4	0.591
**Body-mass index (kg/m^2^)**	23.8±3.5	23.5±3.1	23.6±2.3	0.424
**Current smoker**	62 (10.4%)	22 (11.8%)	3 (11.5%)	0.857
**Hypertension**	424 (70.9%)	150 (80.2%)	20 (76.9%)	0.039
**Diabetes mellitus**	197 (32.9%)	71 (38.0%)	10 (38.5%)	0.406
**Hypercholesterolemia**	177 (29.6%)	66 (35.3%)	9 (34.6%)	0.314
**Previous MI**	33 (5.5%)	15 (8.0%)	3 (11.5%)	0.250
**Previous PCI**	92 (15.4%)	32 (17.1%)	2 (7.7%)	0.453
**Previous CABG**	7 (1.2%)	3 (1.6%)	1 (3.8%)	0.486
**Previous CVA**	61 (10.2%)	27 (14.4%)	3 (11.5%)	0.277
**Congestive heart failure**	36 (6.0%)	16 (8.6%)	0 (0.0%)	0.186
**Chronic kidney disease**	40 (6.7%)	9 (4.8%)	4 (15.4%)	0.119
**Familial history of CAD**	16 (2.7%)	11 (5.9%)	1 (3.8%)	0.110
**Anemia**	283 (47.3%)	88 (47.1%)	11 (42.3%)	0.882
**Presentation with ACS**	335 (56.0%)	104 (55.6%)	13 (50.0%)	0.832
**PRECISE-DAPT score**	26.1 ± 9.7	25.3 ± 8.7	26.0 ± 9.2	0.564
**- High risk**	276 (46.2%)	89 (47.6%)	13 (50.0%)	0.541
**CYP2C19**				0.268
**- NM**	224 (37.5%)	68 (36.4%)	6 (23.1%)	
**- IM**	295 (49.3%)	85 (45.5%)	16 (61.5%)	
**- PM**	79 (13.2%)	34 (18.2%)	4 (15.4%)	

Values are expressed as the number of patients (%) or mean (SD). ACS, acute coronary syndrome; CABG, coronary artery bypass graft; CAD, coronary artery disease; CVA, cerebrovascular accident; IM, intermediate metabolizer; MI, myocardial infarction; NM, normal metabolizer; PCI, percutaneous coronary intervention; PM, poor metabolizer.

### Clinical outcome

**Figure 1:**
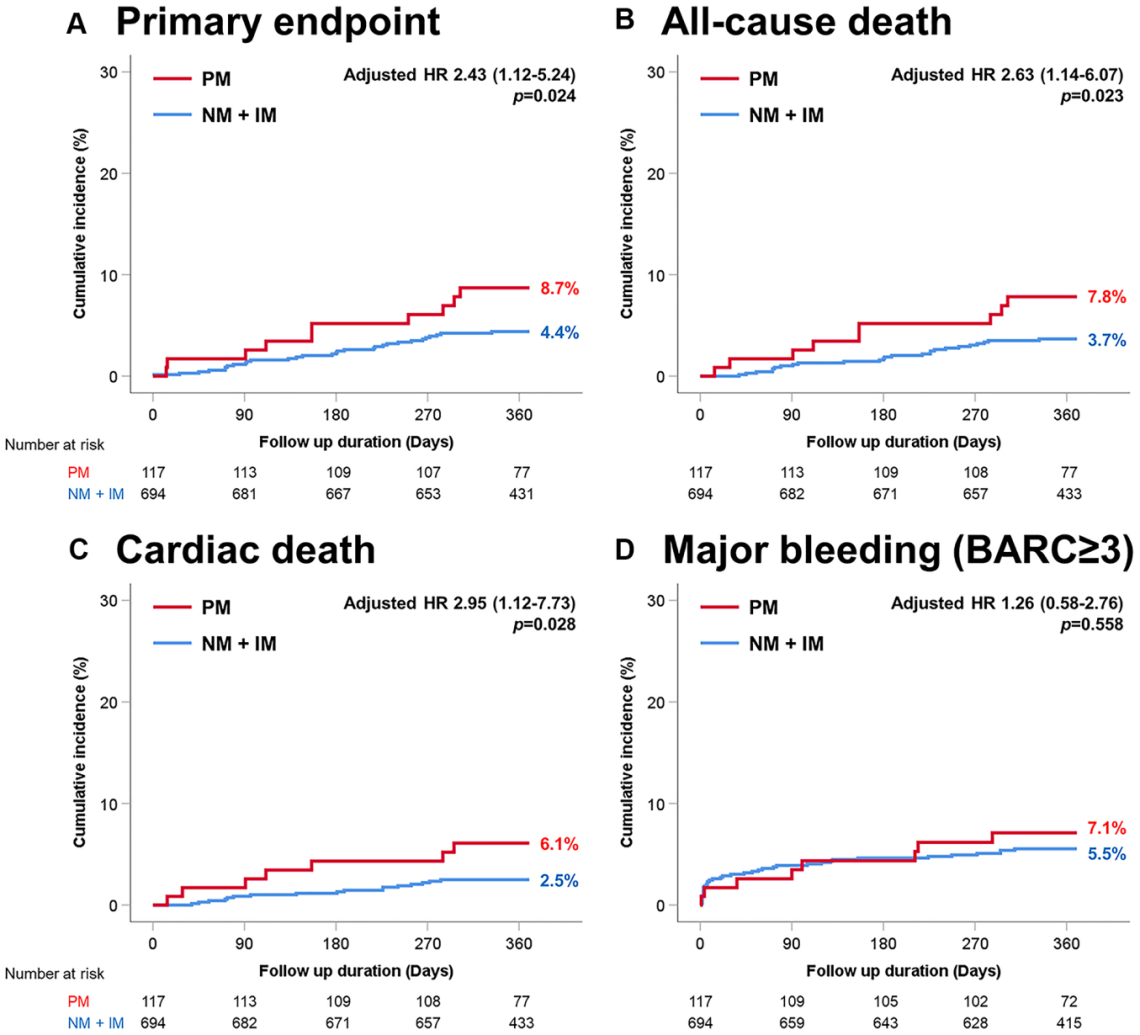
**Time-to-event curves through 1-year for selected adverse events according to CYP2C19 variant.** (**A**) Primary endpoint (myocardial infarction and death). (**B**) All-cause death. (**C**) Cardiac death. (**D**) Major bleeding (BARC≥3). PM, poor metabolizer; NM, normal metabolizer; IM, intermediate metabolizer.

For CYP2C19, the primary endpoint, defined as the composite of myocardial infarction and death, was significantly higher in the poor metabolizer group than in the other group (PM vs NM or IM; 8.7% vs. 4.4%; Figure 1). Regarding the secondary endpoints, death and cardiac death were more common in the poor metabolizer group than in the other group. There were no differences in myocardial infarction, stent thrombosis, target lesion revascularization, stroke, or major bleeding between the two groups (Table 3).

**Table 3:** Cumulative incidence of clinical outcomes according to CYP2C19 variant in elderly patients.

	**NM or IM**	**PM**
	**(n=694)**	**(n=117)**
**Primary endpoint**	30 (4.4%)	10 (8.7%)
**Any Death**	25 (3.7%)	9 (7.8%)
**Cardiac Death**	17 (2.5%)	7 (6.1%)
**Myocardial infarction**	7 (1.0%)	2 (1.7%)
**Stent thrombosis**	7 (1.0%)	3 (2.6%)
**Target lesion revascularization**	32 (4.6%)	6 (5.1%)
**Major bleeding**	38 (5.5%)	8 (7.1%)

Values are expressed as the number of patients (%: cumulative incidence). The primary endpoint was a composite of myocardial infarction and death.

**Figure 2:**
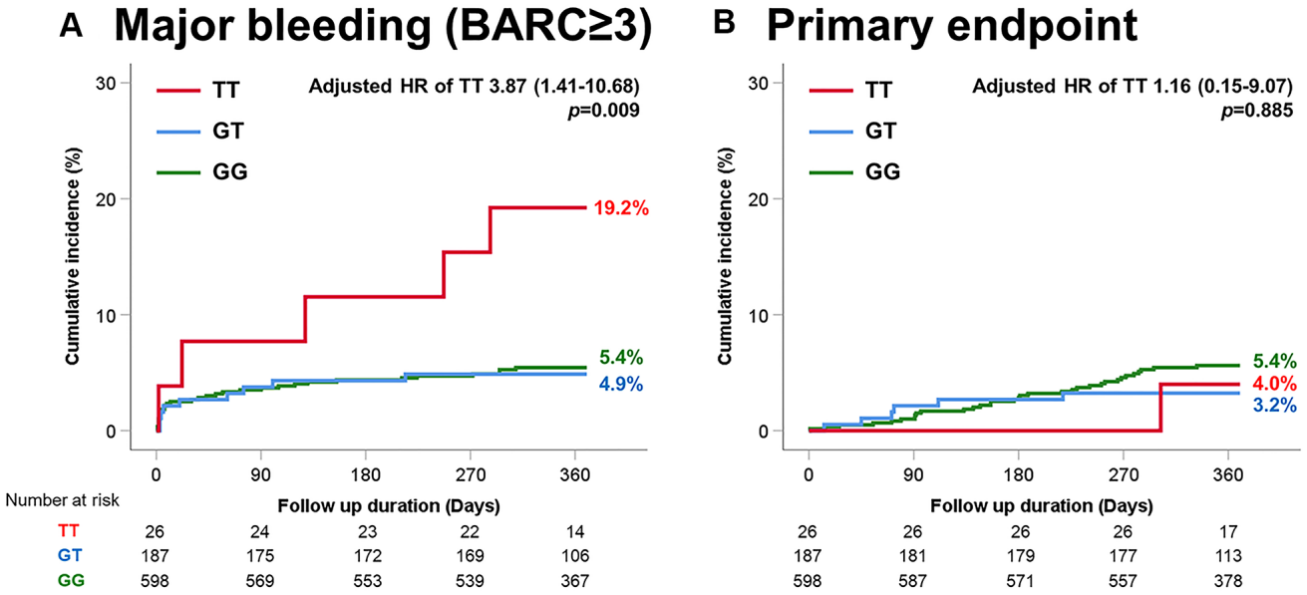
**Time-to-event curves through 1-year for selected adverse events according to P2Y12 G52T variant.** (**A**) Major bleeding (BARC≥3). (**B**) Primary endpoint (myocardial infarction and death).

For the P2Y12 platelet ADP receptor gene G52T polymorphism, there were no significant differences in ischemic events, including primary endpoint between the groups (Table 4). However, the TT group had a significantly higher occurrence rate of major bleeding than the other groups (TT vs. GT vs. GG; 19.2% vs. 4.9% vs. 5.4%; Figure 2).

**Table 4:** Cumulative incidence of clinical outcomes according to P2Y12 platelet adenosine 5′-diphosphate receptor G52T polymorphism in elderly patients.

	**GG**	**GT**	**TT**
	**(N=598)**	**(N=187)**	**(N=26)**
**Primary endpoint**	33 (5.4%)	6 (3.2%)	1 (4.0%)
**Any Death**	27 (4.5%)	6 (3.2%)	1 (3.8%)
**Cardiac Death**	20 (3.3%)	4 (2.1%)	0 (0.0%)
**Myocardial infarction**	8 (1.3%)	1 (0.5%)	0 (0.0%)
**Stent thrombosis**	7 (1.2%)	3 (1.6%)	0 (0.0%)
**Target lesion revascularization**	25 (4.2%)	11 (5.9%)	2 (7.7%)
**Major bleeding**	32 (5.4%)	9 (4.8%)	5 (19.2%)

Values are expressed as the number of patients (%: cumulative incidence). The primary endpoint was a composite of myocardial infarction and death.

### Genetic variation to adverse clinical outcome

**Figure 3:**
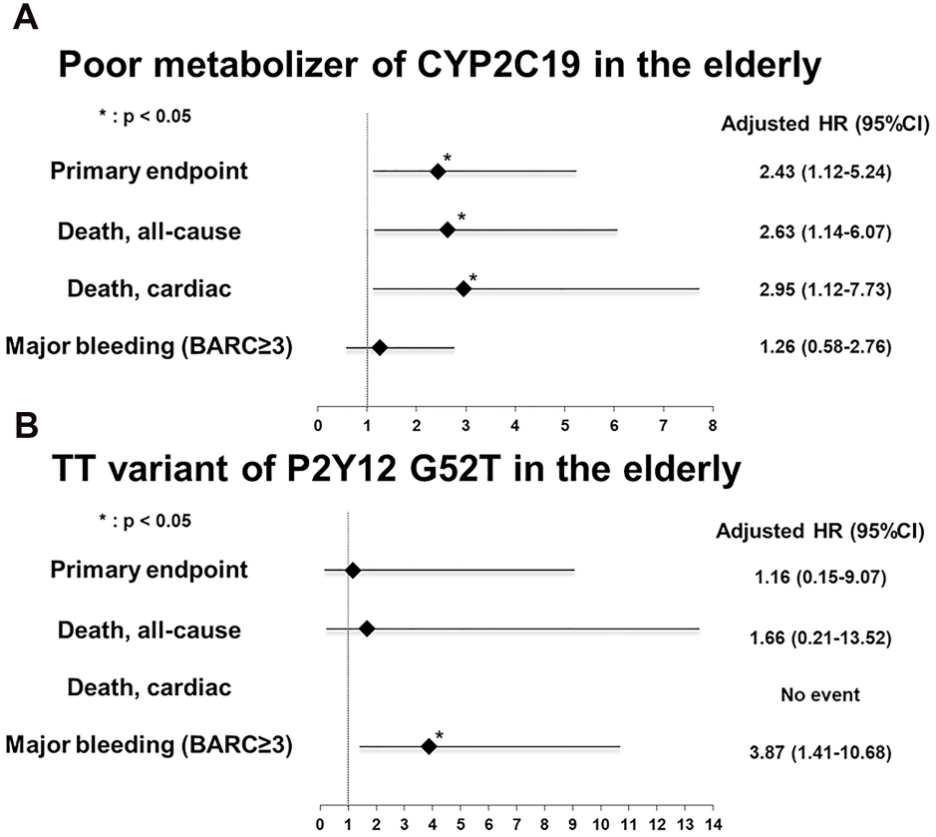
**Adjusted multivariate risk of genetic variant in the elderly for subsequent one-year adverse events.** (**A**) Poor metabolizer of CYP2C19 (**B**) TT of P2Y12 platelet adenosine 5′-diphosphate receptor gene G52T polymorphism. Hazard ratio was adjusted for sex, hypertension, diabetes mellitus, previous history of myocardial infarction, previous history of percutaneous coronary intervention, congestive heart failure, chronic kidney disease, current smoking status, anemia, clinical presentation to acute coronary syndrome, genomic variations (CYP2C19, P2Y12, PON1, and ABCB1), duration of dual antiplatelet therapy, multivessel involvement, minimal stent size, and total stent length. BARC, Bleeding Academic Research Consortium; HR, hazard ratio.

Poor metabolizers of CYP2C19 had a higher risk for the primary endpoint than the other group (hazard ratio [HR] 2.43; 95% confidence interval [95% CI] 1.12–5.24; p=0.024; Figure 3). In addition, poor metabolizers were a prognostic factor of death (HR 2.63; 95% CI 1.14–6.07; p=0.023; Figure 3) and cardiac death (HR 2.95; 95% CI 1.12–7.73, p=0.028; Figure 3). Chronic kidney disease (HR 4.73; 95% CI 2.13–10.52, p<0.001) and current smoking (HR 2.62; 95% CI 1.08–6.34, p=0.033), and presentation with acute coronary syndrome (HR 2.60; 95% CI 1.17–5.81, p=0.020) were independent prognostic factors for the primary endpoint. Otherwise, a longer duration of DAPT resulted in more protective results (HR 0.991; 95% CI 0.988–0.993, p<0.001).

Regarding major bleeding, the TT variant of P2Y12 G52T (HR 3.87, 95% CI 1.41–10.68, p=0.009; Figure 3) and anemia (HR 2.39; 95% CI 1.24–4.61, p=0.009) were independent predictors. Meanwhile, poor metabolizers of the CYP2C19 variant was no statistical significance in major bleeding (HR 1.26; 95% CI 0.58–2.76, p=0.558).

## DISCUSSION

With the increasing rates of PCI in elderly patients, the increase in ischemic and bleeding events due to DAPT in this patient group needs to be addressed. However, the current guidelines offer little evidence on how to determine the optimal DAPT for the elderly [11, 12]. Moreover, studies on tailored DAPT based on genetic information in elderly patients are lacking. Our results suggest that identifying genetic variations in elderly patients helps guide DAPT treatment in this patient group undergoing PCI.

The main findings of this study were as follows: 1) CYP2C19 and P2Y12 platelet ADP receptor gene G52T polymorphism affect clinical outcomes in elderly patients. 2) Regarding CYP2C19, the poor metabolizers had a higher risk of death, cardiac death, and composite events of myocardial infarction and death even after adjusting for other genetic and clinical variables. 3) Regarding P2Y12 G52T, the TT group had a higher risk of major bleeding when adjusting for other genetic and clinical variables.

Loss-of-function CYP2C19 alleles decrease the active metabolites of clopidogrel in the blood, thereby reducing the inhibitory effect of clopidogrel on platelet aggregation [13]. Many studies have shown that loss-of-function CYP2C19 alleles are associated with a higher risk of worse clinical outcomes [14–16]. In addition, the U.S. Food and Drug Administration reported a drug safety concern for poor metabolizers of the CYP2C19 variant [17]. However, routine genetic testing in clinical practice is excluded from the current guidelines. The Consortium Guidelines recommend different clopidogrel treatments for patients with loss-of-function CYP2C19 alleles [10]. Briefly, potent P2Y12 inhibitors, such as ticagrelor, are helpful in poor metabolizers. However, in the case of intermediate metabolizers or dose adjustment for clopidogrel, the results are controversial [18]. Moreover, few studies have been conducted on drug adjustment according to genetic variations in the elderly. Thus, we investigated the impact of CYP2C19 variants on clinical outcomes after PCI in elderly patients.

This study showed that elderly patients with poor metabolizers, which require adjusting the antiplatelet therapy, had worse clinical outcomes than the normal and intermediate metabolizer groups when the same DAPT treatment (aspirin and clopidogrel) was applied. However, a longer duration of DAPT was protective against ischemic events in multivariate analysis, and there were no differences in major bleeding between the two groups. Thus, our results suggest that an adjusted dose of potent P2Y12 inhibitor replacement benefits poor metabolizers in elderly patients despite concern for maintaining a long duration of DAPT.

Recent studies, including POPular Genetics and TAILOR-PCI, reported that a genotype-guided oral P2Y12 inhibitor strategy did not significantly reduce ischemic events compared with standard clopidogrel therapy in patients with CYP2C19 loss-of-function [8, 19]. However, the risk of ischemic events in patients with DAPT increases with age [20]. Changes in platelet aggregation, which may contribute to increased ischemic events, increase as patient age increases, that is, a higher rate of ischemic events occurs due to increased platelet aggregability in the elderly [21]. Regarding the relationship between age and genomic variation, Dücker et al. [22] reported that systemic exposure might be increased in elderly patients, even if their old age affects pharmacokinetics and they share the same genetic polymorphism. The authors reported that additional systemic exposure of approximately 1.5-fold occurred by age, even in the same poor metabolizer group, and more clinical events could occur [22]. This might explain why comparisons of clinical outcomes among the poor, normal, and intermediate metabolizers yielded negative results in the general population analysis [8, 23]. Our results revealed that worse clinical outcomes might occur in elderly patients who are poor metabolizers, as the platelet dysfunction caused by biological senescence from old age and the intrinsic risk factors caused by genetic polymorphisms interact with each other. The mechanism by which these interactions operate is not yet known, and further research is required.

P2Y12, which is a G protein-coupled receptor, plays a crucial role in platelet aggregation and is activated by ADP [24, 25]. The P2Y12 receptor is a target of clopidogrel. The P2Y12 platelet ADP receptor gene G52T is one of the polymorphisms in exon 2 of the P2Y12 gene, as reported by Fontana [26]. Despite the low incidence in the TT group and a lack of evidence of the correlation between the TT variant and clinical outcomes, the TT variant was investigated as a potential marker of the P2Y12 gene. Recently, an association between the P2Y12 G52T variant and bleeding events has been reported [27]. Thus, we investigated the impact of P2Y12 G52T in elderly patients. Our results revealed that the risk of major bleeding was higher in the TT group than in the other groups. Patient who received DAPT in the TT group may have a higher probability of hemorrhage. Our results suggest that elderly patients with the TT variant benefit from adjusting the duration of DAPT to prevent major bleeding.

Elderly patients have high ischemia and bleeding risks. The recent definition of the Academic Research Consortium for High Bleeding Risk (ARC-HBR) identifies advanced age (>75 years) as a bleeding risk factor [28, 29]. In addition, the PRECISE-DAPT score also includes age among the five items that predict bleeding risk [30, 31]. Thus, proper risk stratification for bleeding risk and ischemic risk for elderly patients should be conducted. As the aforementioned studies reviewed, the rate of bleeding risk based on the PRECISE-DAPT was high in this study, which was conducted for the elderly. The average PRECISE-DAPT score of patients enrolled in this study was 25.9±9.4, and 46.6% of patients were included in the high-risk cluster. However, we found no difference in the PRECISE-DAPT score and rate of the high-risk cluster according to CYP2C19 and P2Y12 variants; however, major bleeding events occurred frequently in the TT group. Thus, the probability of bleeding appears to vary according to gene variants, even in elderly patients. Thus, genotype testing might benefit elderly patients treated with DAPT after PCI.

Although evidence-based studies in elderly patients are required, clinical trials in the elderly have not been conducted as often as in younger patients. Even in the current guidelines, the optimal DAPT for elderly patients is unclear because of numerous variables that potentially impact outcomes [11, 12]. A recent study reported changes in ischemia and bleeding in the elderly according to the duration of DAPT [32]. These results suggest that platelet function or genetic tests guided P2Y12 inhibitor adjustment may lead to better clinical outcomes in the elderly. To the best of our knowledge, our study is the first study to show that adjusting the treatment policy according to genetic variation among elderly patients undergoing PCI should be considered for better clinical outcomes. Importantly, our study revealed clinical outcomes after a year of follow-up after PCI. A longer-term investigation may provide insight into the clinical impact of the genotype according to clopidogrel maintenance or discontinuation. Since aspirin and clopidogrel were used as DAPT in this study, the impact on the clinical outcomes of the potent inhibitor ticagrelor is insufficient. Therefore, our results should be interpreted with caution. Finally, we cannot exclude the effects of other drugs, which were not investigated, that affect the metabolism of CYP2C19.

In conclusion, in elderly patients who maintained standard DAPT after PCI, poor metabolizers of CYP2C19 had poor clinical outcomes regarding death and myocardial infarction. The TT group of P2Y12 G52T had a higher risk of major bleeding.

## MATERIALS AND METHODS

### Study population

This subanalysis study included 811 elderly patients (≥75 years of age) from a prospective registry (GENIUS study, NCT02707445) (Supplementary Figure 1). The GENIUS study included 5000 patients who underwent PCI for coronary artery disease in 20 tertiary hospitals and investigated the influence of genotyping on coronary artery stenting outcomes between February 2012 and July 2014. The inclusion criteria were as follows: 1) age >20 years and 2) all percutaneous coronary intervention patients within 1 month. Patients who had an allergy to aspirin or clopidogrel, pregnancy, or a life expectancy <1 year were excluded from this registry. Among the 5000 patients, 413 patients were excluded for various reasons, such as inclusion/exclusion criteria violation, follow-up loss, consent withdrawal, missing genotyping results, and missing platelet function test results. In addition, 98 patients with rapid metabolizers (*17) in CYP2C19 were excluded from the analysis because of their confounding effects [33]. Thus, among the 4489 patients, 811 patients aged ≥75 years were defined as elderly patients. DAPT was recommended for a duration of 1 year (3 months) after the index PCI. DAPT included aspirin (100 mg daily) and clopidogrel (75 mg daily). Other P2Y12 inhibitors, such as ticagrelor and prasugrel, and anticoagulants were not prescribed after PCI. The study protocol was approved by the institutional review board of each participating center. Written informed consent was obtained from all patients at enrollment. This study complied with the Declaration of Helsinki and was registered with ClinicalTrials.gov (NCT02707445).

### Laboratory tests

Measured single nucleotide polymorphisms were CYP2C19*2 (rs4244285), CYP2C19*3 (rs4986893), CYP2C19*17 (rs12248560), ABCB1 (rs1045642), PON1 (rs662), and P2Y12 G52T (rs6809699). The genotype of each single nucleotide polymorphism was determined by pyrosequencing using a PSQ 96MA Pyrosequencer (Pyrosequencing AB, Uppsala, Sweden), as previously reported [34]. The results for ABCB1 and PON1 are described in the supplemental materials (Supplementary Figure 2 and Supplementary Tables 6, 7). The VerifyNow P2Y12 assay (Accumetrics, San Diego, CA, USA) was used to measure the inhibitory effect of clopidogrel on platelet reactivity. The results are reported as PRUs. The residual platelet reactivity and genotype results were blinded to the physicians and patients.

### Definitions

The primary endpoint were myocardial infarction and death. Secondary endpoints were an individual event of any death, cardiac death, myocardial infarction, stent thrombosis, target lesion revascularization, stroke, and major bleeding defined as the Bleeding Academic Research Consortium (BARC 3, 4, and 5 [35].

### Statistics

Between-group comparisons were performed using the independent Student’s t-test or analysis of variance for continuous variables and chi-square tests for categorical variables. Post hoc subgroup analysis was performed in accordance with the baseline characteristics. To estimate the effect of clinical outcomes, including myocardial infarction, death, cardiac death, stent thrombosis, target lesion revascularization, stroke, and major bleeding according to genetic variation, the HR was calculated using the Cox proportional hazards model. In the multivariate Cox regression analysis, HR was adjusted for sex, hypertension, diabetes mellitus, previous history of myocardial infarction, previous history of PCI, congestive heart failure, chronic kidney disease, current smoking status, anemia, clinical presentation to acute coronary syndrome (unstable angina, non-ST elevation myocardial infarction, and ST-elevation myocardial infarction), genetic variants (CYP2C19, P2Y12 G52T, PON1, and ABCB1), the duration of DAPT, multivessel involvement, minimal stent size, and total stent length. Two-tailed p-values were used, and p-values <0.05, were considered statistically significant. All analyses were performed using SPSS version 25.0 software (SPSS Inc., Chicago, IL, USA).

## Supplementary Material

Supplementary Figures

Supplementary Tables
