# Supplementary material for: Impact of genetic variants on clinical outcome after percutaneous coronary intervention in elderly patients
Source: Aging (Albany NY). 2021 Mar 12;13(5):6506–24. doi: 10.18632/aging.202799 (PMC7993709; doi:10.18632/aging.202799)
Supplement: Supplementary Figures [file aging-13-202799-s001.pdf]

SUPPLEMENTARY FIGURES

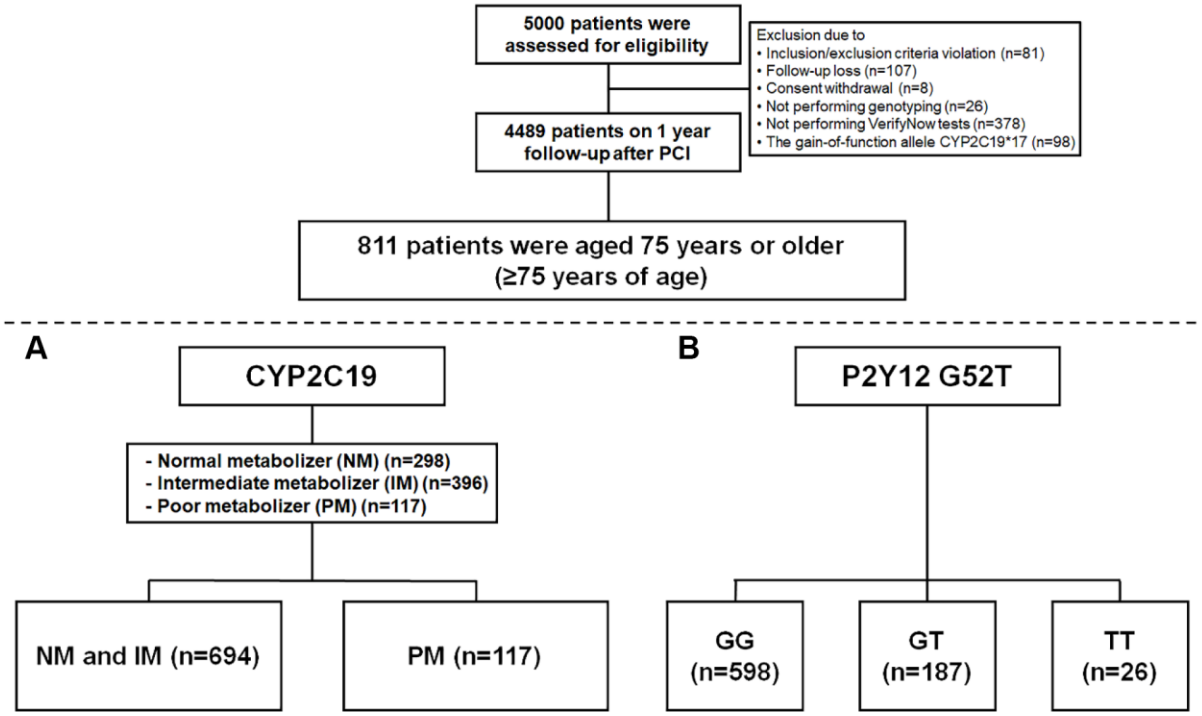

Supplementary Figure 1. Flow Chart (**A**) CYP2C19. (**B**) P2Y12 platelet adenosine 5′-diphosphate receptor gene G52T polymorphism.

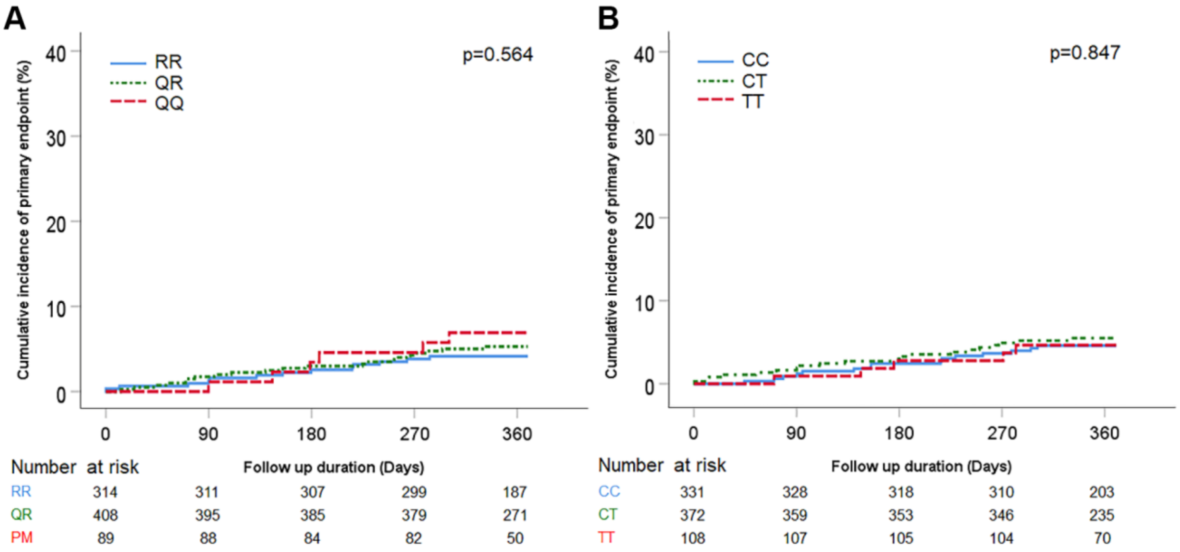

Supplementary Figure 2. Time-to-event curves through 1-year for primary endpoint according to PON1 and ABCB1. (**A**) PON1 (**B**) ABCB1.
