# Supplementary material for: Impact of genetic variants on clinical outcome after percutaneous coronary intervention in elderly patients
Source: Aging (Albany NY). 2021 Mar 12;13(5):6506–24. doi: 10.18632/aging.202799 (PMC7993709; doi:10.18632/aging.202799)
Supplement: Supplementary Tables [file aging-13-202799-s002.pdf]

SUPPLEMENTARY TABLES

Supplementary Table 1. Baseline characteristics and genomic variation in elderly patients (≥75 years of age).

	Total (n=811)
**Age (years)**	79.1±3.6
**Male sex**	433 (53.3%)
**Body-mass index (kg/m^2)**	23.7±3.4
**Hypertension**	594 (73.2%)
**Diabetes mellitus**	278 (34.3%)
**Hypercholesterolemia**	252 (31.1%)
**Previous MI**	51 (6.3%)
**Previous PCI**	126 (15.5%)
**Previous CABG**	11 (1.4%)
**Previous CVA**	91 (11.2%)
**Congestive heart failure**	52 (6.4%)
**Chronic kidney disease**	53 (6.5%)
**Familial history of CAD**	28 (3.5%)
**Presentation with ACS**	452 (55.7%)
**CYP2C19**	
**- Normal metabolizer (*1/*1)**	298 (36.7%)
**- Intermediate metabolizer**	396 (48.8%)
***1/*2**	292 (36.0%)
***1/*3**	104 (12.8%)
**- Poor metabolizer**	117 (14.4%)
***2/*2**	66 (8.1%)
***2/*3**	40 (4.9%)
***3/*3**	11 (1.4%)
**P2Y12 G52T (rs6809699)**	
**- GG**	598 (73.7%)
**- GT**	187 (23.1%)
**- TT**	26 (3.2%)

Data are number of patients (%) or mean (SD). ACS, acute coronary syndrome; CABG, coronary artery bypass graft; CAD, coronary artery disease; CVA, cerebrovascular accident; PCI, percutaneous coronary intervention.

Supplementary Table 2. Other genetic variations (PON1 and ABCB1) and lesion characteristics according to CYP2C19 variant in elderly patients (≥75 years of age).

	NM or IM (N=694)	PM (N=117)	p
**PON1**			0.738
**- RR**	266 (38.3%)	48 (41.0%)	
**- QR**	353 (50.9%)	55 (47.0%)	
**- QQ**	75 (10.8%)	14 (12.0%)	
**ABCB1**			0.678
**- CC**	287 (41.4%)	44 (37.6%)	
**- CT**	314 (45.2%)	58 (49.6%)	
**- TT**	93 (13.4%)	15 (12.8%)	
**Multivessel disease**	111 (16.0%)	21 (17.9%)	0.693
**Left anterior descending artery**	400 (57.6%)	79 (67.5%)	0.056
**Left circumflex artery**	157 (22.6%)	26 (22.2%)	1.000
**Right coronary artery**	250 (36.0%)	35 (29.9%)	0.240
**Left main**	31 (4.5%)	7 (6.0%)	0.630
**Exist of visible thrombus**	30 (4.3%)	5 (4.3%)	1.000
**Thrombosuction**	33 (4.8%)	2 (1.7%)	0.210
**Number of stents**			0.275
**- 1**	518 (74.6%)	82 (70.1%)	
**- 2**	136 (19.6%)	25 (21.4%)	
**- 3 or over**	40 (5.8%)	10 (8.6%)	
**Number of lesions**	1.4 ± 1.1	1.6 ± 1.3	0.197
**Minimal stent size**	2.9 ± 0.4	2.8 ± 0.4	0.072
**Total length of stent**	31.3 ± 17.6	33.4 ± 21.6	0.319

Data are number of patients (%) or mean (SD).

Supplementary Table 3. In-hospital event, discharge medication, P2Y12 reaction unit, and dual antiplatelet therapy duration according to CYP2C19 variant in elderly patients (≥75 years of age).

	NM or IM (N=694)	PM (N=117)	*P*-value
**In-hospital event**			
**Death**	0 (0.0%)	1 (0.9%)	0.311
**TLR**	0 (0.0%)	0 (0.0%)	-
**Myocardial infarction**	6 (0.9%)	0 (0.0%)	0.670
**Stent thrombosis**	0 (0.0%)	0 (0.0%)	-
**Stroke**	2 (0.3%)	0 (0.0%)	1.000
**Bleeding**	30 (4.3%)	6 (5.1%)	0.882
**Discharge medication**			
**Aspirin**	690 (99.4%)	116 (99.1%)	1.000
**Clopidogrel**	685 (98.7%)	117 (100.0%)	0.446
**Cilostazol**	43 (6.2%)	10 (8.5%)	0.453
**Proton pump inhibitor**	147 (21.2%)	24 (20.5%)	0.967
**CCB**	196 (28.2%)	36 (30.8%)	0.653
**Statin**	646 (93.1%)	110 (94.0%)	0.863
**ARB**	270 (38.9%)	52 (44.4%)	0.303
**ACEi**	172 (24.8%)	26 (22.2%)	0.631
**BB**	402 (57.9%)	76 (65.0%)	0.184
**Platelet function test**			
**VerifyNow PRU**	234.0±74.8	269.9±82.8	<0.001
**VerifyNow inhibition**	25.0±25.6	17.7±38.2	0.049
**Duration of DAPT**			
**Total duration (days)**	322.7±87.1	313.7±103.0	0.373
**>6 months**	600 (86.5%)	99 (84.6%)	0.697
**>12 months**	569 (82.0%)	97 (82.9%)	0.913

Data are number of patients (%) or mean (SD). ACEi, angiotensin-converting-enzyme inhibitor; ARB, angiotensin II receptor blocker; BB, beta blocker; CCB, calcium channel blocker; DAPT, dual antiplatelet therapy; IM, intermediate metabolizer; NM, normal metabolizer; PM, poor metabolizer; PRU, P2Y12 reaction unit; TLR, target lesion revascularization.

Supplementary Table 4. Other genetic variations (PON1 and ABCB1) and lesion characteristics according to P2Y12 G52T polymorphism in elderly patients (≥75 years of age).

	GG (N=598)	GT (N=187)	TT (N=26)	p
**PON1**				0.486
**- RR**	231 (38.6%)	71 (38.0%)	12 (46.2%)	
**- QR**	304 (50.8%)	95 (50.8%)	9 (34.6%)	
**- QQ**	63 (10.5%)	21 (11.2%)	5 (19.2%)	
**ABCB1**				0.881
**- CC**	245 (41.0%)	75 (40.1%)	11 (42.3%)	
**- CT**	276 (46.2%)	86 (46.0%)	10 (38.5%)	
**- TT**	77 (12.9%)	26 (13.9%)	5 (19.2%)	
**Multivessel disease**	91 (15.2%)	39 (20.9%)	2 (7.7%)	0.092
**Left anterior descending artery**	355 (59.4%)	107 (57.2%)	17 (65.4%)	0.699
**Left circumflex artery**	128 (21.4%)	47 (25.1%)	8 (30.8%)	0.338
**Right coronary artery**	204 (34.1%)	78 (41.7%)	3 (11.5%)	0.006
**Left main**	29 (4.8%)	9 (4.8%)	0 (0.0%)	0.517
**Exist of visible thrombus**	30 (5.0%)	3 (1.6%)	2 (7.7%)	0.093
**Thrombosuction**	30 (5.0%)	3 (1.6%)	2 (7.7%)	0.093
**Number of stents**				0.793
**- 1**	451 (75.4%)	129 (69.0%)	20 (76.9%)	
**- 2**	111 (18.6%)	45 (24.1%)	5 (19.2%)	
**- 3 or over**	36 (6.0%)	13 (6.9%)	1 (3.9%)	
**Number of lesions**	1.5 ± 1.2	1.4 ± 0.9	1.1 ± 0.3	0.285
**Minimal stent size**	2.9 ± 0.4	2.9 ± 0.4	2.9 ± 0.3	0.750
**Total length of stent**	31.0 ± 17.5	34.0 ± 20.5	29.9 ± 16.0	0.125

Data are number of patients (%) or mean (SD).

Supplementary Table 5. In-hospital event, discharge medication, P2Y12 reaction unit, and dual antiplatelet therapy duration according to P2Y12 G52T polymorphism in elderly patients (≥75 years of age).

	GG (N=598)	GT (N=187)	TT (N=26)	*P*-value
**In-hospital event**				
**Death**	1 (0.2%)	0 (0.0%)	0 (0.0%)	0.837
**TLR**	0 (0.0%)	0 (0.0%)	0 (0.0%)	-
**Myocardial infarction**	5 (0.8%)	1 (0.5%)	0 (0.0%)	0.828
**Stent thrombosis**	0 (0.0%)	0 (0.0%)	0 (0.0%)	-
**Stroke**	0 (0.0%)	1 (0.5%)	1 (3.8%)	<0.001
**Bleeding**	27 (4.5%)	4 (2.1%)	5 (19.2%)	<0.001
**Discharge medication**				
**Aspirin**	593 (99.2%)	187 (100.0%)	26 (100.0%)	0.408
**Clopidogrel**	591 (98.8%)	185 (98.9%)	26 (100.0%)	0.854
**Cilostazol**	36 (6.0%)	14 (7.5%)	3 (11.5%)	0.449
**Proton pump inhibitor**	129 (21.6%)	35 (18.7%)	7 (26.9%)	0.536
**CCB**	169 (28.3%)	53 (28.3%)	10 (38.5%)	0.528
**Statin**	556 (93.0%)	175 (93.6%)	25 (96.2%)	0.799
**ARB**	239 (40.0%)	73 (39.0%)	10 (38.5%)	0.966
**ACEi**	145 (24.2%)	50 (26.7%)	3 (11.5%)	0.236
**BB**	352 (58.9%)	107 (57.2%)	19 (73.1%)	0.305
**Platelet function test**				
**VerifyNow PRU**	238.4±77.2	239.4±78.5	254.2±60.8	0.593
**VerifyNow inhibition**	24.4±30.2	23.6±20.5	16.5±15.5	0.363
**Duration of DAPT**				
**Total duration (days)**	319.4±91.0	324.4±89.4	346.5±39.1	0.280
**> 6 months**	506 (84.6%)	163 (87.2%)	25 (96.2%)	0.203
**> 12 months**	467 (78.1%)	156 (83.4%)	22 (84.6%)	0.233

Data are number of patients (%) or mean (SD). ACEi, angiotensin-converting-enzyme inhibitor; ARB, angiotensin II receptor blocker; BB, beta blocker; CCB, calcium channel blocker; DAPT, dual antiplatelet therapy; PRU, P2Y12 reaction unit; TLR, target lesion revascularization.

Supplementary Table 6. Clinical presentation, duration of DAPT, PRU, and in-hospital event according to PON1 variant.

	RR (N=314)	QR (N=408)	QQ (N=89)	p
**Presentation with ACS**	177 (56.4%)	222 (54.4%)	53 (59.6%)	0.649
**Duration of DAPT**				
**Total duration (days)**	329.4 ± 78.0	315.2 ± 98.1	322.2 ± 85.3	0.106
**> 12 months**	259 (82.5%)	315 (77.2%)	71 (79.8%)	0.219
**Platelet function test**				
**VerifyNow PRU**	237.5 ± 81.3	239.3 ± 74.2	244.6 ± 74.5	0.743
**In-hospital event**				
**Death**	0 (0.0%)	1 (0.2%)	0 (0.0%)	0.610
**TLR**	0 (0.0%)	0 (0.0%)	0 (0.0%)	-
**Myocardial infarction**	3 (1.0%)	2 (0.5%)	1 (1.1%)	0.696
**Stent thrombosis**	0 (0.0%)	0 (0.0%)	0 (0.0%)	-
**Stroke**	0 (0.0%)	2 (0.5%)	0 (0.0%)	0.372
**Bleeding**	11 (3.5%)	20 (4.9%)	5 (5.6%)	0.564

ACS, acute coronary syndrome; DAPT, dual antiplatelet therapy; TLR, target lesion revascularization; PRU, P2Y12 reaction unit.

Supplementary Table 7. Clinical presentation, duration of DAPT, PRU, and in-hospital event according to ABCB1 variant.

	CC (N=331)	CT (N=372)	TT (N=108)	p
**Presentation with ACS**	175 (52.9%)	212 (57.0%)	65 (60.2%)	0.332
**Duration of DAPT**				
**Total duration (days)**	320.0 ± 88.9	321.9 ± 92.3	324.3 ± 82.4	0.902
**> 12 months**	258 (77.9%)	302 (81.2%)	85 (78.7%)	0.554
**Platelet function test**				
**VerifyNow PRU**	239.2 ± 75.6	242.5 ± 77.3	227.5 ± 79.6	0.205
**In-hospital event**				
**Death**	0 (0.0%)	1 (0.3%)	0 (0.0%)	0.554
**TLR**	0 (0.0%)	0 (0.0%)	0 (0.0%)	-
**Myocardial infarction**	3 (0.9%)	2 (0.5%)	1 (0.9%)	0.826
**Stent thrombosis**	0 (0.0%)	0 (0.0%)	0 (0.0%)	-
**Stroke**	1 (0.3%)	1 (0.3%)	0 (0.0%)	0.854
**Bleeding**	15 (4.5%)	15 (4.0%)	6 (5.6%)	0.791

ACS, acute coronary syndrome; DAPT, dual antiplatelet therapy; TLR, target lesion revascularization; PRU, P2Y12 reaction unit.
